# Risk Factors for the Progression of Mild Cognitive Impairment in Different Types of Neurodegenerative Disorders

**DOI:** 10.1155/2018/6929732

**Published:** 2018-06-05

**Authors:** Pei-Hao Chen, Shih-Jung Cheng, Hui-Chi Lin, Chuo-Yu Lee, Chih-Ho Chou

**Affiliations:** ^1^Department of Neurology, MacKay Memorial Hospital, Taipei, Taiwan; ^2^Department of Medicine, Mackay Medical College, New Taipei City, Taiwan; ^3^Graduate Institute of Mechanical and Electrical Engineering, National Taipei University of Technology, Taipei, Taiwan; ^4^Department of Physical Therapy and Assistive Technology, National Yang-Ming University, Taipei, Taiwan; ^5^Graduate Institute of Chemistry, Tamkang University, New Taipei City, Taiwan; ^6^Department of Neurology, Chi-Mei Medical Center, Tainan, Taiwan; ^7^Chia Nan University of Pharmacy and Science, Tainan, Taiwan

## Abstract

**Objective:**

Mild cognitive impairment (MCI) is a transitional state between normal aging and early dementia. It has a heterogeneous etiology and clinical course. This study aimed to examine the factors associated with the progression of MCI in different types of dementia disorders.

**Method:**

A retrospective, longitudinal, observational study of outpatients with MCI was conducted at a medical center in northern Taiwan. Patient medical records were reviewed, and risk factors were analyzed by multivariate analysis.

**Results:**

Among 279 patients with MCI, 163 (58.4%), 68 (24.4%), and 48 (17.2%) were diagnosed with Alzheimer's disease, vascular cognitive impairment, and Lewy body diseases, respectively. During the observation period, 37.2% of patients progressed to dementia. Older age and a higher Clinical Dementia Rating Scale-Sum of Boxes were associated with the risk of progression. Hyperlipidemia was associated with a decreased risk. Converters were more likely to receive an antidementia prescription.

**Conclusion:**

Our study suggests the importance of comprehensive clinical profiling, risk factor assessment, and detailed drug history evaluations in improving our understanding and management of dementia subtypes.

## 1. Introduction

Dementia can result from several underlying diseases, including Alzheimer's disease (AD), cerebrovascular disease, Lewy body diseases (LBD), frontotemporal dementia, and other less common disorders such as Huntington's disease, supranuclear palsy, and others. Mild cognitive impairment (MCI) is considered an intermediate state between normal cognitive aging and very early dementia. Individuals diagnosed with MCI may remain stable, return to normal (14.4–55.6% of patients), or progress to dementia [1]. There is extensive literature on MCI in AD but limited to other types of dementia. Many epidemiological studies have reported that the presence of vascular risk factors (i.e., hypertension, diabetes, cerebrovascular disease, and hyperlipidemia) in midlife is associated with an increased risk of cognitive impairment and dementia, particularly AD and vascular dementia (VaD) [2, 3]. Previous studies have reported older age, lower education status, prestroke cognitive and functional status, and history of diseases as risk factors for poststroke dementia [4]. However, cardiovascular risk factors in the midlife increase the risk of dementia, but their roles in the late-life are less clear [5]. Apart from vascular risk factors, other clinical conditions have been reported to show associations with MCI and dementia, including chronic obstructive pulmonary disease [6], chronic heart disease [7], cirrhosis [8], and chronic kidney disease [9]. By contrast, there has been a limited focus on whether chronic comorbid illnesses exhibit differential associations for different types of dementia. Cognitive impairment, seen with both delirium and dementia, has been associated with polypharmacy. Inappropriate use of benzodiazepine (BZD) in the elderly is also a major public health problem. The consequences of inappropriate BZD use include falls, delirium, other cognitive dysfunction, acute respiratory failure, car accidents, dependence, and withdrawal symptoms [10]. Studies on associations between sedative hypnotics and cognitive decline in elderly patients have yielded mixed findings [11]. Previous studies cannot yet determine whether the observed epidemiological association is a causal effect or the result of unmeasured confounding variables.

It is important to understand which clinical and medical factors might be associated with cognitive decline. Identifying these risk factors that hasten the onset of dementia is crucial for timely medical intervention and predicting prognoses. Improved knowledge of comorbidities in patients with MCI would facilitate the development of preventive strategies aimed at slowing rapid clinical and functional deterioration. This study aimed to examine the association between common clinical and neuropsychological factors in later life at the MCI stage and the risk of converting to dementia, with a particular focus on specific dementia disorders.

### 1.1. Specific

#### 1.1.1. Study Design

This was a retrospective, longitudinal, observational study that used an unselected sample to test for associations between comorbidities in patients with MCI and the rate of cognitive decline or dementia.

#### 1.1.2. Setting

Data used for this present study were obtained from a dementia care database from January 2014 to June 2017.

#### 1.1.3. Participants

**Figure 1:**
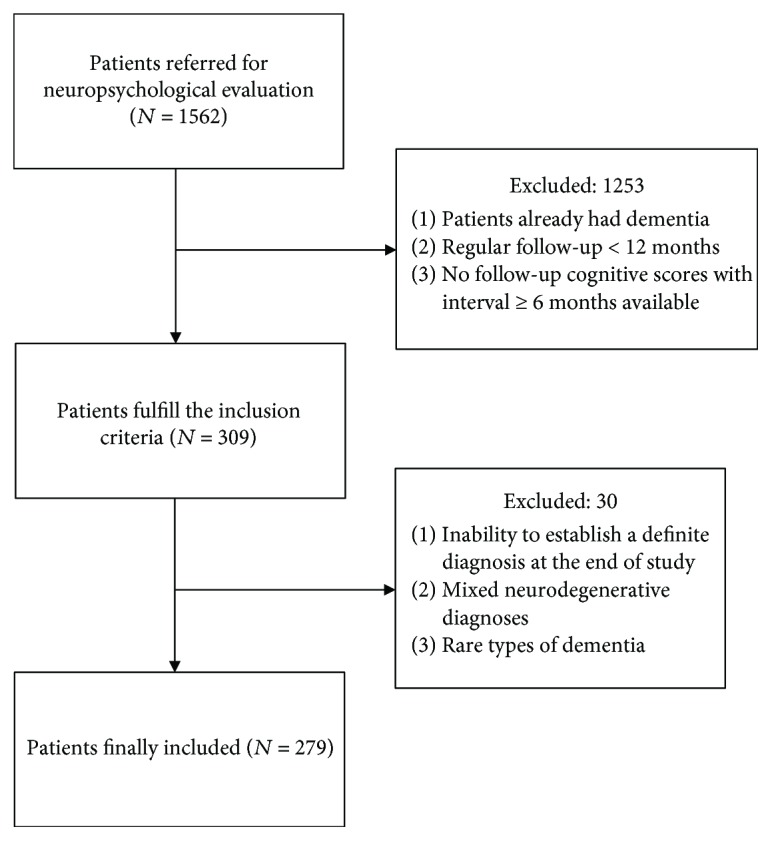
Flow diagram showing the patient exclusion criteria.

Patients were enrolled and studied at the neurological department of the MacKay Memorial Hospital (Taiwan). We recruited patients by reviewing electronic medical records and documenting information related to clinical measurements, diagnoses, comorbidities, neuroimaging reports, biochemical tests, and neuropsychological assessments at the first visit. The medical records were reevaluated by a dementia expert who was not involved in the assessments of the patients. The rate of decline in cognition was measured based on changes in the Mini-Mental Status Exam (MMSE) scores [12] and Clinical Dementia Rating Scale-Sum of Boxes (CDR-SB) [13]. A previous study reported that the conversion to dementia from MCI stages could be predicted by MMSE changes over time instead of single measurements [14]. This approach is useful for the detection of AD and other dementias in people with MCI. The Clinical Dementia Rating Scale (CDR) was initially designed to stage clinical dementia in older persons [15]. It yields both a global score and CDR-SB score. The global score is used to stage dementia severity, whereas the CDR-SB score is a more detailed quantitative version of that scale [16]. Progression of MCI to dementia (converter) was defined as a change in the overall CDR score from 0.5 to ≥1.0. The inclusion criteria were as follows: (1) cognitive complaints not normal for the age of the individual, (2) no dementia (cut-off MMSE score > 23 in persons with at least 6 years of education or >13 in persons with less than 6 years of education and CDR = 0.5), (3) cognitive decline, and (4) essentially normal functional activities in everyday life at the first visit [17]. All participants were evaluated from the first visit and followed up at regular intervals for at least one year until a clinical diagnosis of specific dementia (converter) or MCI (nonconverter) was reached. We included patients who had received regular ambulatory care for 12 months or longer. The intervals between two cognitive assessments were greater than six months. Exclusion criteria were as follows: (1) inability to establish a definite diagnosis for converters at the end of the study; (2) clinical suspicion of frontotemporal dementia, corticobasal degeneration, progressive supranuclear palsy, or other rare types of dementia; and (3) mixed dementia or mixed neurodegenerative diagnoses. In cases of atypical clinical manifestations, one optimal diagnosis for each patient was made under the consensus of neurologists. We excluded patients with uncertain dementia syndromes.  Figure 1 provides a flowchart of the inclusion/exclusion criteria.

#### 1.1.4. Classification of Dementia Syndromes

Diagnoses of dementia syndromes were confirmed by two study clinicians who reviewed clinical, neuropsychological, and brain imaging data and biochemical tests. Comprehensive neuropsychological testing was performed by an experienced clinical psychologist. Dementia subtypes were classified using international consensus clinical criteria. Dementia due to AD was diagnosed in patients who fulfilled the National Institute of Neurological and Communicative Disorders and Stroke and the Alzheimer's Disease and Related Disorders Association criteria for probable AD [18]. Dementia or MCI due to vascular disease was diagnosed in patients who fulfilled the American Heart Association/American Stroke Association Statement on Vascular Contributions to Cognitive Impairment and Dementia criteria [19]. LBD is an umbrella term for two closely related clinical diagnoses: Parkinson's disease dementia (PDD) and dementia with Lewy bodies (DLB). The third report of the DLB Consortium was used for probable DLB diagnoses [20]. The diagnosis of PDD was made based on recommendations of the movement disorder society task force [21]. The onset age of dementia was defined as the date on which the clinical symptoms and neuropsychological tests first allowed the diagnosis to be made. For nonconverters, MCI due to AD was diagnosed based on the National Institute on Aging and the Alzheimer's Association work group criteria [22]. Neuroimaging criteria for vascular cognitive disorders were based on the 2014 International Society for Vascular Behavioral and Cognitive Disorders (VASCOG) statement [23]. Diagnostic criteria for MCI due to Parkinson's disease or DLB are based on international consensus clinical criteria [24, 25].

#### 1.1.5. Comorbidities and Medication History

Comorbidities, including diabetes mellitus, hypertension, hypercholesterolemia, heart disease (history of coronary artery disease, atrial fibrillations, heart failure, or valvular heart disease), gastrointestinal disorder, chronic kidney disease, liver function impairment, anemia, and depression, were documented by reviewing the medical records and laboratory documents in the hospital. Medication history of using antidementia agents (cholinesterase inhibitors or memantine) and hypnotic agents, including BZD or nonbenzodiazepine hypnotic agents (“Z drugs”) during observational periods, were identified from the medical records and/or medical history interviews. Taking five or more regular medications prescribed >30 days at the time of diagnosis was defined as polypharmacy in our study.

#### 1.1.6. Statistical Methods

We used a stepwise multiple logistic regression model to analyze the data. Each categorical variable was examined using *χ*^2^ tests. The Student *t*-test was used to analyze continuous variables. Variables with a *p* value ≤ 0.1 were included in the multiple regression model. The effects of each variable were represented by odds ratios and corresponding 95% confidence intervals (CIs), which were calculated based on the exponential coefficient of the multiple logistic regression model. We also analyzed these factors in different etiologic subgroups. All statistical analyses were performed using R version 3.4.2 (2017-09-28).

#### 1.1.7. Ethical Review

This present study was reviewed and approved by the MacKay Memorial Hospital Institutional Review Board (number 18MMHIS037).

## 2. Results

**Figure 2:**
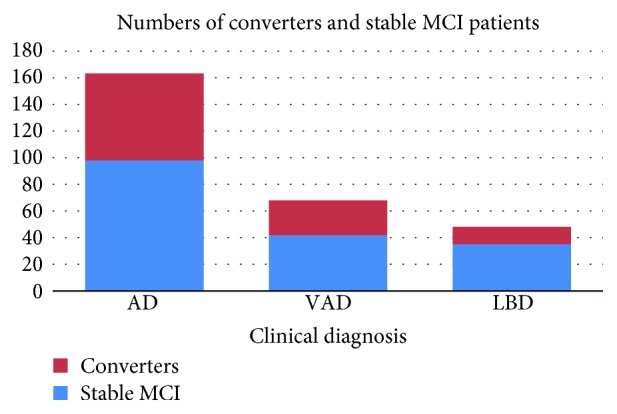
Proportions of conversion to dementia in patients with different clinical diagnoses. AD: Alzheimer's disease; VaD: vascular dementia; LBD: Lewy body diseases.

We recruited 279 patients with MCI from the database. A total of 163 patients were diagnosed with AD (female 64.4%), 68 had vascular cognitive impairment (female 30.9%), and 48 had Lewy body diseases (LBD) (female 45.8%). The distributions of age did not differ significantly among these groups. In this patient cohort, 58.7%, 31.8%, and 46.5% had hypertension, diabetes, and hypercholesterolemia, respectively, while 19.2% diagnosed as having depressive disorders. Additionally, 37.4% of our MCI patients progressed to dementia with a mean follow-up period of 27.09 ± 15.09 months. The proportion of conversion in the three dementia syndromes was 39.9% in AD, 38.2% in VaD, and 27.1% in LBD (*p* = 0.2683) (Figure 2). Univariate tests showed that the ages of onset, CDR-SB, and baseline MMSE were significant between stable MCI and converters (Table 1). Converters were more likely to receive antidementia agents (66.3% versus 34.9%, *p* < 0.001). The MMSE scores could be divided into subscores for orientation, memory, calculation, language, and perceptual motor function. The subscores of orientation were significantly different between MCI converters and nonconverters.

A multiple logistic regression model revealed that older age at onset, female sex, and a greater CDR-SB were significantly associated with a higher risk of converting from MCI to dementia. Receiving antidementia agents was strongly associated with a higher probability of this conversion (Table 2). Hyperlipidemia was associated with a decreased risk of conversion. The interaction between hyperlipidemia and etiologies was nonsignificant. We also evaluated baseline MMSE scores (substituting CDR-SB in the model), and the results of the other risk factors were unaltered. We tested four interactions between etiologies and each one of DM, hypertension, stroke, and heart disease, respectively, in the multiple logistic regression model, and all were nonsignificant. These interactions were not reported. Selection of risk factors in the model was based on the previous studies and aforementioned statistical criteria.

We performed a subgroup analysis from the MCI cohort according to the etiology. Age of onset was a significant risk factor in AD and VaD. Female was associated with an increased risk in AD. CDR-SB was associated with higher risk in AD and VaD. Receiving antidementia agents was significantly associated with conversion in AD. The trend was mostly consistent with the finding in the main cohort (Table 3). The LBD and AD subgroups had very similar risk factor profiles of conversion from MCI to dementia.

## 3. Discussion

MCI is heterogeneous in its etiology and clinical course. Older age and higher CDR-SB are major important markers for the identification of patients at a higher risk of progression. In our present study, we found that CDR-SB is highly indicative both in the whole cohort and different subgroup analyses. Because of the scoring rule, a CDR global score of 0.5 was unable to detect the cognitive categories that were affected (i.e., memory, orientation, judgment/problem solving, community affairs, hobbies, and personal care). In contrast, CDR-SB includes all categories, and the sensitivity might differ from the CDR global score.

Cholesterol has been recently shown to be important for synaptic transmission, and many neurodegenerative diseases, including AD and PD, are characterized by impaired cholesterol turnover in the brain [26]. A population-based prospective cohort study from Denmark showed that subjects with objective cognitive impairment who were most likely to progress were older, physically inactive, had a higher level of total cholesterol, and had a history of depression [27]. Reviews and meta-analyses revealed that midlife high total serum cholesterol was associated with an increased risk of MCI, AD, and cognitive decline in late-life; however, high cholesterol in late-life was not associated with MCI, AD, VaD, any dementia, or cognitive decline [28]. Patients with late-life cardiovascular factors, including body mass index, atrial fibrillation, hypertension, hyperlipidemia, and diabetes, were more likely to have a diagnosis of VaD than the normal populations. However, there were no associations between AD and DLB with hypertension, hyperlipidemia, or diabetes [5]. In our study, we found that hypercholesterolemia was not a risk factor but had a nonsignificant protective effect on the progression of MCI. More than 70% of our patients used statins during the study period. However, we were not sure based on the prescription pattern, whether these patients received treatment from other hospitals or clinics. A previous study concluded that statins may slow the rate of cognitive decline and delay the onset of AD and all-cause dementia in cognitively healthy elderly individuals, whereas individuals with MCI may not have comparable cognitive protection from these agents [29]. A meta-analysis of two large trials, including a total of 26,340 patients aged ≥ 40 years with cardiovascular risk factors, reported that statins administered in later life do not prevent cognitive decline or dementia [30].

In Taiwan, cholinesterase inhibitors and memantine can be provided by the health insurance system under the following rules. AD or PDD was diagnosed by a neurologist or psychiatrist using the clinical diagnostic criteria. They had the latest MMSE scores of 10–26 and did not have contraindications. The authority required annual reevaluations of the initial prescription, and the reimbursement would be discontinued in case there is too much deterioration in MMSE scores or CDR. In our study, 45.5% of patients received antidementia agents during the observation period. Prescribing these medicines was associated with the likelihood of converting to dementia. However, those who received antidementia treatments also had higher CDR-SB (significant) and lower baseline MMSE scores (not significant). A possible explanation is that the physicians more likely prescribed antidementia agents to improve the cognitive functions in patients who they found were more severe at baseline. The results did not imply that the clinical courses in MCI patients were changed by these treatments.

Risks of cognitive decline and delirium were known to be associated with polypharmacy. In a prospective cohort study of 294 elderly patients, 22% taking five or fewer medications were found to have impaired cognition when compared with 33% of patients taking 6–9 medications and 54% in patients taking 10 or more medications [31]. A previous study of patients with cognitive impairment found that 70.4% were on multiple medications and 42% took BZD [32]. In our study, 61.5% of patients took more than five types of medications and almost 40% of patients were prescribed sedative/hypnotic drugs for at least 30 days during the study. In a large cohort of postmenopausal women, all types of antidepressant use and different levels of depression severity were associated with subsequent cognitive impairment [33]. By contrast, MCI has reported being a risk factor for depressive and anxiety disorders, suggesting common pathological pathways for cognitive and psychiatric outcomes [34]. A recently published article found that executive dysfunction in elderly people with depression may be associated with the age effect [35]. The reciprocal effects of depression and MCI were inconclusive. Our data revealed a nonsignificant trend (*p* = 0.06) supporting a link between depression and cognitive decline.

The effects of hypnotic agents and risk of dementia remained a subject of debate. A recent study based on longitudinal data found a slight association, but no dose-effect, in elderly nondemented individuals [36]. We found that hypnotic agents were very commonly used in depressed patients in our cohort. Neither depression nor hypnotics use was associated with conversion from MCI to dementia. These findings are consistent with a recent study performed in Taiwan [37].

### 3.1. Limitations

In our study, the diagnosis of dementia syndromes was justified by two clinicians who were experienced in dementia care. Comprehensive neuropsychological testing was performed by an experienced clinical psychologist. We excluded MCI of uncertain etiology and mixed neurodegenerative diagnoses. Lack of pathological confirmation was a weakness of our study, similar to the majority of the previously published articles. The primary clinical etiologic diagnosis remained stable in more than 90% of our patients. Previous results from a multicenter longitudinal database suggested that hospital-based studies were more accurate in categorizing dementia subtypes because they involve a multidisciplinary diagnostic approach and an adequate diagnostic infrastructure compared with community-based studies [38]. Due to the Personal Data Protection Act of 2010 of Taiwan, medication history may not be perfectly completed. The small case numbers limited our capacity to analyze whether there were any differences among different dementia syndromes.

## 4. Conclusions

Our retrospective, longitudinal, observational study showed the distribution of dementia subtypes from MCI and described the clinical progression, comorbidity, and medication history associated with dementia subtypes. We believe that comprehensive clinical profiling, assessments of risk factors, and detailed drug history evaluations are helpful to better understand and manage dementia subtypes. Our study suggests the importance of early detection of individuals with MCI. In the future, prospective studies should establish which of these factors are the most influential to aid the development of treatment and prevention strategies. Polypharmacy and hypnotic sedatives should be avoided if they are not essential, especially in elderly patients with MCI.

## Figures and Tables

**Table 1:** Univariate analysis of clinical characteristics in 279 MCI patients who remained stationary course or progressed to dementia.

	Stable MCI	Dementia	*p* value
	*N* = 175 (percentage)	*N* = 104 (percentage)	
Etiology	AD 98 (56)	AD 65 (62.5)	0.268
	VaD 42 (24)	VaD 26 (25)	
	LBD 35 (20)	LBD 13 (12.5)	
Age of onset	70.9	74.7	<0.001
Female	100 (57.1)	48 (46.2)	0.098
Education	6.9	7.3	0.425
CDR-SB	2.20	2.96	<0.001
MMSE	24.5	23.3	0.004
Hypertension	101 (57.7)	63 (60.6)	0.954
DM	51 (29.1)	38 (36.5)	0.373
Dyslipidemia	90 (51.4)	39 (37.5)	0.021
Liver disease	24 (13.7)	19 (18.3)	0.279
Renal disease	20 (11.4)	14 (13.5)	0.844
Gastrointestinal disease	82 (46.9)	50 (48.1)	0.942
Anemia	10 (5.7)	7 (6.7)	0.918
Heart disease	73 (41.7)	47 (45.2)	0.894
Stroke	43 (24.6)	25 (24)	1.000
Depression	26 (14.9)	25 (24)	0.065
Antidementia agents	61 (34.9)	69 (66.3)	<0.001
Hypnotic agents	61 (34.9)	49 (47.1)	0.064
Polypharmacy	103 (58.9)	69 (67.0)	0.314

AD: Alzheimer's disease; VaD: vascular dementia; LBD: Lewy body diseases; MMSE: Mini-Mental State Examination; CDR-SB: Clinical Dementia Rating-Sum of Boxes; DM: diabetes mellitus. Remark: antidementia agents: acetylcholinesterase inhibitors and memantine. Hypnotic agents: benzodiazepines and nonbenzodiazepine hypnotics.

**Table 2:** Logistic regression model of variables associated with conversion to dementia in the MCI cohort.

	OR	2.50%	97.50%	*p* value
(Intercept)	0.0014	1.00*E* − 04	0.029	<0.001
VaD	2.082	0.910	4.909	0.089
LBD	0.536	0.225	1.223	0.146
Age	1.071	1.032	1.113	<0.001
Gender	0.540	0.293	0.980	0.044
CDR-SB	1.553	1.221	2.008	<0.001
Hyperlipidemia	0.554	0.310	0.980	0.044
Depression	1.624	0.730	3.623	0.233
Antidementia agents	5.162	2.663	10.522	<0.001
Hypnotic agents	1.346	0.719	2.517	0.352

VaD: vascular dementia; LBD: Lewy body diseases; CDR-SB: Clinical Dementia Rating-Sum of Boxes. Remark: antidementia agents: acetylcholinesterase inhibitors and memantine. Hypnotic agents: benzodiazepines and nonbenzodiazepine hypnotic agents.

**Table:** (a) Subgroup of AD

		OR	2.50%	97.50%	*p* value
(Intercept)		0.008	1.00*E* − 04	0.309	0.013
Age		1.058	1.010	1.112	0.021
Gender		0.424	0.202	0.874	0.021
CDR-SB		1.395	1.030	1.913	0.034
Hyperlipidemia		0.761	0.364	1.569	0.462
Depression		1.124	0.419	2.985	0.814
Antidementia agents		3.411	1.572	7.789	0.003
Hypnotic agents		1.800	0.808	4.051	0.151

**Table:** (b) Subgroup of LBD

	OR	2.5%	97.50%	*p* value
(Intercept)	0	0	0.529	0.067
Age	1.186	1.021	1.446	0.051
Gender	0.143	0.012	1.009	0.080
CDR-SB	1.079	0.486	2.358	0.845
Dyslipidemia	0.321	0.045	1.825	0.218
Depression	7.221	0.720	106.364	0.109
Antidementia agents	66.025	7.839	1340.956	0.001
Hypnotic agents	0.410	0.057	2.3819	0.337

**Table:** (c) Subgroup of VaD

	OR	2.50%	97.5%	*p* value
(Intercept)	0.000	0.000	0.068	0.009
Age	1.106	1.030	1.203	0.010
Gender	0.563	0.129	2.233	0.421
CDR-SB	2.172	1.229	4.309	0.015
Hyperlipidemia	0.459	0.131	1.550	0.211
Depression	8.786	1.059	101.545	0.057
Hypnotic agents	0.820	0.216	2.934	0.762

AD: Alzheimer's disease; VaD: vascular dementia; LBD: Lewy body diseases; CDR-SB: Clinical Dementia Rating-Sum of Boxes. Remark: hypnotic agents indicate benzodiazepines and nonbenzodiazepine hypnotic agents. Antidementia agents were not reimbursed for VaD patients in Taiwan health insurance system. They were not analyzed in the model due to sparsity.

## Data Availability

The data used to support the findings of this study are available from the corresponding author upon request.
